# Structural Basis and Catalytic Mechanism for the Dual Functional Endo-β-N-Acetylglucosaminidase A

**DOI:** 10.1371/journal.pone.0004658

**Published:** 2009-03-02

**Authors:** Jie Yin, Lei Li, Neil Shaw, Yang Li, Jing Katherine Song, Wenpeng Zhang, Chengfeng Xia, Rongguang Zhang, Andrzej Joachimiak, Hou-Cheng Zhang, Lai-Xi Wang, Zhi-Jie Liu, Peng Wang

**Affiliations:** 1 National Laboratory of Biomacromolecules, Institute of Biophysics, Chinese Academy of Sciences, Beijing, China; 2 Graduate School of the Chinese Academy of Sciences, Beijing, China; 3 National Glycoengineering Research Center and The State Key Laboratory of Microbial Technology, School of Life Sciences, Shandong University, Shandong, China; 4 Departments of Biochemistry and Chemistry, The Ohio State University, Columbus, Ohio, United States of America; 5 Structural Biology Center, Advanced Photon Source, Argonne National Laboratory, Illinois, United States of America; 6 Institute of Human Virology and Department of Biochemistry and Molecular Biology, University of Maryland School of Medicine, Baltimore, Maryland, United States of America; 7 College of Pharmacy and The State Key Laboratory of Elemento-Organic Chemistry, Nankai University, Tianjin, China; University of Queensland, Australia

## Abstract

Endo-β-*N*-acetylglucosaminidases (ENGases) are dual specificity enzymes with an ability to catalyze hydrolysis and transglycosylation reactions. Recently, these enzymes have become the focus of intense research because of their potential for synthesis of glycopeptides. We have determined the 3D structures of an ENGase from *Arthrobacter protophormiae* (Endo-A) in 3 forms, one in native form, one in complex with Man_3_GlcNAc-thiazoline and another in complex with GlcNAc-Asn. The carbohydrate moiety sits above the TIM-barrel in a cleft region surrounded by aromatic residues. The conserved essential catalytic residues – E173, N171 and Y205 are within hydrogen bonding distance of the substrate. W216 and W244 regulate access to the active site during transglycosylation by serving as “gate-keepers”. Interestingly, Y299F mutation resulted in a 3 fold increase in the transglycosylation activity. The structure provides insights into the catalytic mechanism of GH85 family of glycoside hydrolases at molecular level and could assist rational engineering of ENGases.

## Introduction

**Figure 1:**
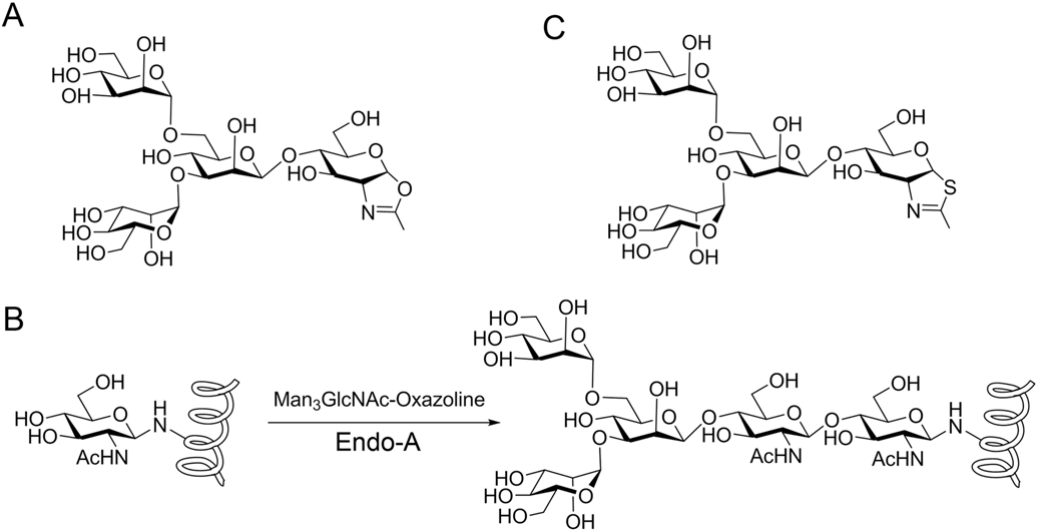
Synthesis of glycopeptides using oligosaccharide oxazolines as sugar donors. (A) Mimic of oxazoline ion intermediate, Man_3_GlcNAc-oxazoline. (B) Endo-A catalyzed glycopeptide synthesis by using Man_3_GlcNAc-oxazoline. (C) An Endo-A inhibitor, Man_3_GlcNAc-thiazoline.

Endo-β-*N*-acetylgucosaminidases (EC 3.2.1.96) (ENGases) are a class of enzymes that hydrolyze the glycosidic bond - GlcNAc β-1,4GlcNAc - present in *N*-linked sugar chains in glycoproteins and release the *N*-glycan moiety. These enzymes are key enzymes in the processing event of free oligosaccharides in the cytosol [1]. ENGases are widely distributed in bacteria [2]–[4], fungi [5], [6], plants [7], [8] and animals [9], [10]. According to amino acid sequence similarity, the enzymes are classified into glycoside hydrolase families GH18 and GH85 [11]. Besides hydrolysis, some ENGases of GH85 family possess transglycosylation activity, i.e., the ability to transfer the released oligosaccharide moiety to a suitable acceptor other than water. For example, Endo-A can transfer a high-mannose type oligosaccharide to monosaccharides such as *N*-acetylglucosamine (GlcNAc) and glucose to form a new oligosaccharide [2], [11], whereas the ENGase from *Mucor hiemalis* (Endo-M), exhibits significant transglycosylation activity towards complex type oligosaccharides [12]. When the acceptor is a pre-assembled GlcNAc-containing peptide or protein, Endo-A and Endo-M are able to transfer an intact oligosaccharide to the acceptor to form a new glycopeptide or glycoprotein in a single step, making it a highly convergent chemoenzymatic approach [13]–[15]. However, ENGases are inherently a class of hydrolases. The transglycosylation activities in general are relatively low in comparison with their hydrolytic activity, particularly when natural *N*-glycan or natural glycopeptide is used as the donor substrate. Moreover, product hydrolysis is a major issue for this chemoenzymatic approach, as the product thus formed would turn out to be the substrate of the enzyme. Recently, exploration of synthetic sugar oxazolines (Figure 1A) [16], the mimics of the presumed enzymatic reaction intermediate, as the donor substrates has not only broadened the availability of donor substrates, but also significantly enhanced the overall efficiency of the chemoenzymatic synthesis [17]. A number of homogeneous natural and unnatural *N*-glycopeptides and glycoproteins were synthesized by this chemoenzymatic approach (Figure 1B) [16], [18], [19]. It was shown that the slightly modified *N*-glycan oxazoline could serve as a substrate for transglycosylation, but the resulting *N*-glycopeptide or *N*-glycoprotein, with a truncated or structurally modified *N*-glycan would become resistant to enzymatic hydrolysis due to the slight modification. Nevertheless, product hydrolysis is still a problem when this chemoenzymatic approach is applied to the synthesis of glycoproteins carrying natural *N*-glycans, as the ENGases can rapidly hydrolyze the natural *N*-glycans.

One solution to this problem is to create a glycosynthase that lacks hydrolytic activity but retains transglycosylation activity [20], [21]. In case of a retaining glycosidase, a glycosynthase might be generated by mutating the catalytic nucleophile to a non-nucleophilic residue. The resulting mutant enzyme would lack hydrolytic activity but might still accept an activated glycosyl donor such as glycosyl fluoride with an opposite anomeric configuration for transglycosylation [21]. The routinely employed nucleophile substitution strategy that converts a glycoside hydrolase to a glycosynthase cannot be applied to ENGases because catalysis in ENGases proceeds via a substrate-assisted retaining mechanism [16]. Recent mutagenesis and screening of Endo-M has created some promising mutants that showed glycosynthase activity, that is, they could accept sugar oxazoline corresponding to natural *N*-glycan as substrate for transglycosylation, but lacked the activity to hydrolyze the product [17]. But, unfortunately, the absence of a representative structure for GH85 family of enzymes has become a major bottleneck for rationally engineering ENGases with enhanced transglycosylation activities devoid of hydrolytic activity.

In order to delineate the structural basis for the hydrolysis and transglycosylation activities of Endo-A, we have determined the 3D structures of Endo-A in native form and in complex with Man_3_GlcNAc-thiazoline (Fig 1C) and GlcNAc-Asn. Man_3_GlcNAc-thiazoline is an oxazoline mimic in which the anomeric oxygen at the reducing end was replaced with a sulfur atom (Fig 1C). The Man_3_GlcNAc-thiazoline could be regarded as a transition state mimic and was resistant to Endo-A hydrolysis. It was shown to be an inhibitor for Endo-A [22]. Using the structure of binary complexes as a guide, we have successfully engineered the transglycosylation activity of Endo-A. Further, the structural basis and catalytic mechanism for this dual functional Endo-A is discussed.

## Results

### Overall structure of Endo-A

**Figure 2:**
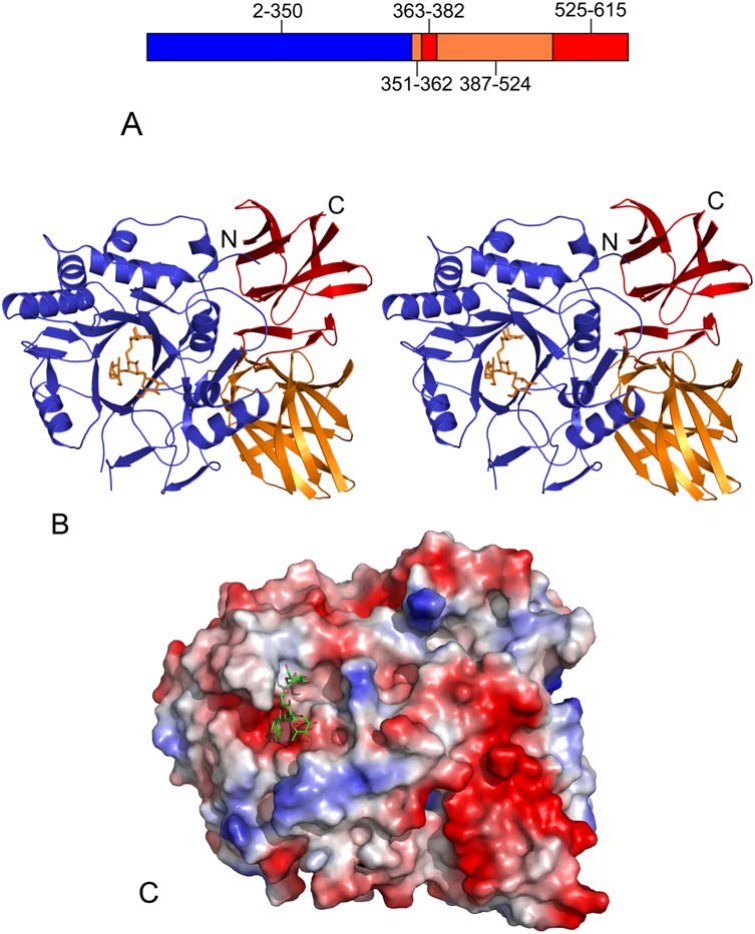
Structure of Endo-A. (A) Diagrammatic representation of Endo-A. Amino acids 1–350 make up Domain 1 (blue), segments 351–362 and 387–524 make up Domain 2 (orange), while segments 363–386 and 525–611 make up Domain 3 (red). (B) Stereo image of Endo-A. Man_3_GlcNAc-thiazoline moiety is shown as sticks. (C) A surface electrostatic potential representation of Endo-A showing the Man_3_GlcNAc-thiazoline moiety sitting inside the active site cleft.

Endo-A from *Arthrobacter protophormiae* is modular in architecture with three distinct domains (Figure 2A). Sequence analysis of Endo-A carried out previously had failed to identify domains 2 and 3 of the enzyme. Domain 1 (Figure 2B), with residues 2–350 being identified, is the largest and displays a typical (β/α)_8_ TIM barrel fold, which has been observed for proteins belonging to family GH18 and GH20 of the glycoside hydrolases. The overall architecture of the domain 1 is similar to that observed in the structure of Endo-F3 [23]. Endo-A has been postulated to carry out substrate hydrolysis and transglycosylation activities using this domain. In addition, the structure of Endo-A reveals two smaller domains, primarily made up of β sheets. The secondary structural elements of these two domains are interdigitated (Figure 2A, 2B). MSD Fold search (http:www.ebi.ac.uk/msd-srv/ssm) for structural homologues of domain 2 and 3 revealed that domain 2 of Endo-A shows similarity to a CBM (carbohydrate binding module, supplementary Figure S1, supplementary Text S1), while the secondary structural elements of domain 3 are similar to Fn3 (fibronectin type III domain, supplementary Figure S2, supplementary Text S1).

**Figure 3:**
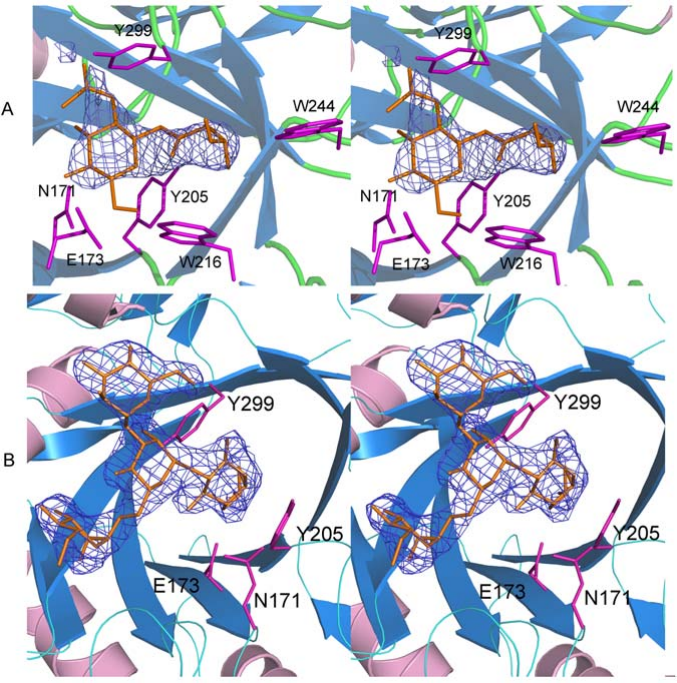
Stereo images of Endo-A in complex with GlcNAc-Asn (panel A) and Man_3_GlcNAc-thiazoline (panel B). The ligand omitted |Fo|-|Fc| α_calc_ electron density map, calculated using a 3.5 Å resolution data set, shown in blue, was contoured at 2.5σ.

In order to determine the structural basis for the binding of the substrate and molecular mechanism for the hydrolysis and transglycosylation activities, we solved the structures of Endo-A in complex with an oxazoline ion intermediate mimic – Man_3_GlcNAc-thiazoline [22] and an acceptor substrate – GlcNAc-Asn. The overall structures of the Endo-A in complex with Man_3_GlcNAc-thiazoline and Endo-A in complex with GlcNAc-Asn are identical to that of the free protein. When compared to the structure of carbohydrate bound Endo-F3, differences are in the loop region surrounding the active site indicating different substrate specificities for the enzymes. Superimposition studies show that the catalytic amino acids occupy similar positions in Endo-A and Endo-F3 structures. The carbohydrate moiety is sitting in a negatively charged cleft region above the β-barrel (Figure 2C). Electron density for the ligands was of good quality and most of the backbone for the GlcNAc-Asn and Man_3_GlcNAc-thiazoline could be traced (Figure 3A, 3B).

### Active site of Endo-A

**Figure 4:**
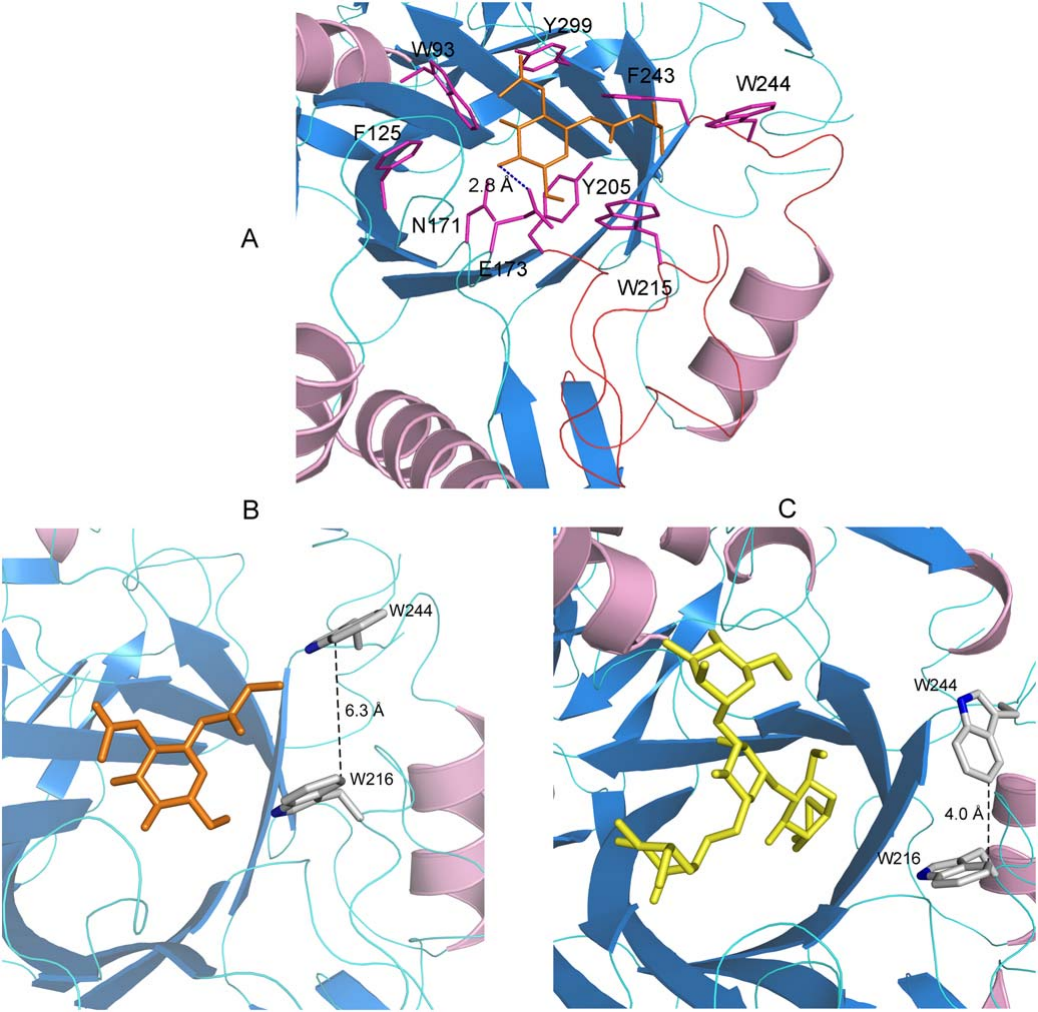
Active site of Endo-A. (A) N171, E173 and Y299 are critical for catalysis. Amino acids surrounding the carbohydrate moiety are shown as sticks. The distances are shown in dashed lines. The GlcNAc-Asn moiety is shown as sticks. (B) W216 and W244 are “gate-keeping” the active site by sterically regulating access to the active site by the acceptor. The side chain of W244 moves during transglycosylation; the distance between the two Trp is much wider and allows passage of an acceptor into the active site. Trp and GlcNAc-Asn are shown as sticks. (C) The gate is “closed” in the structure of free protein (not shown) and Man_3_GlcNAc-thiazoline bound Endo-A (shown as sticks).

The carbohydrate moiety is sitting above the β-barrel and the critical catalytic residues – N171, E173 and Y205 [17]– are hydrogen bonded to GlcNAc-Asn. A number of other residues – W93, F125, W216, F243, W244 and Y299 are interacting with GlcNAc-Asn (Figure 4A). Two loops, comprising of residues 205–224 and 242–248 respectively (shown in red, Figure 4A), are regulating access of the active site to the acceptor. More specifically, W216 and W244, facing each other, are speculated to serve as gate-keepers for the active site during transglycosylation (Figure 4B). In the structures of the free Endo-A and Endo-A bound to Man_3_GlcNAc-thiazoline (Figure 4C), the side chain of W244 is perpendicular to W216 and introduces a steric block at the entrance of the active site. During transglycosylation, the side chain of W244 moves away from the β barrel, becoming parallel to W216 and makes way for the acceptor to enter the active site (Figure 4B). Mutagenesis studies reported previously have indicated the importance of W216 and W244 in transglycosylation. When W216 was mutated to an arginine, Endo-A lost its ability to carry out transglycosylation [24]. Similarly, W251N mutation in Endo-M (equivalent to W244 in Endo-A) reduced the transglycosylation yields dramatically[17]. The minimum distance between the two tryptophans is 6.3 Å (Figure 4B), which is sufficient to allow the passage of an acceptor. Such features are not seen in the structures of Endo-F and Endo-H, both of which have the ability to carry out hydrolysis but lack transglycosylation activities [25]. W216 and W244 probably play a critical role in recognition and docking of the incoming acceptor.

Endo-A catalyses hydrolysis and transglycosylation via a substrate-assisted mechanism, in which the C2 acetamido group mounts a nucleophilic attack at the anomeric carbon resulting in the formation of an oxazoline ion intermediate. This mechanism has been substantiated in part by the observation that the mimics of the oxazoline ion intermediate, such as Man_3_GlcNAc-thiazoline, are potent inhibitors of Endo-A [22]. In order to obtain information about the transition state, we solved the crystal structure of Endo-A in complex with Man_3_GlcNAc-thiazoline (Figure 3B). While the thiazoline part of the carbohydrate moiety is buried deep inside a hydrophobic pocket formed by W93, F125, Y131, F169, Y205, F240, F243 and Y299, the mannose rings reside on the surface. As expected, the nitrogen atom of the thiazoline is hydrogen bonded to the side chain oxygen atom of N171 (supplementary Figure S3). N171 probably orients the acetamido group for a nucleophilic attack and also stabilizes the transition states.

### Site-directed mutagenesis of critical residues

**Figure 5:**
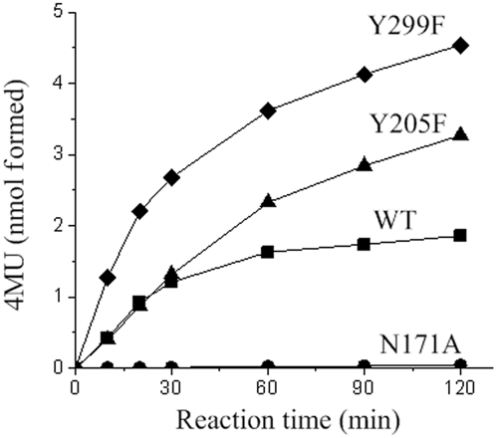
Transglycosylation activity of Endo-A mutants. Time course of transglycosylation reaction of wildtype Endo-A (squares) and the three mutants, N171A (circles), Y205F (triangles), and Y299F (diamonds). The reactions were carried out using a chitinase-coupled assay, in which Man_9_GlcNAc_2_Asn-Fmoc was used as the donor substrate and 4MU-GlcNAc as the acceptor, 0.25 µg of each enzyme was used in a 50 µl system.

In order to probe the role of residues surrounding the carbohydrate moiety in the function of Endo-A, we carried out mutagenesis and performed hydrolysis and transglycosylation assays using the mutants. Using the structures of the binary complexes of Endo-A and results of previous mutagenesis studies of Endo-M [17] as a guide, residues N171, Y205, and Y299 were selected for mutagenesis in order to engineer an Endo-A with diminished hydrolysis activity and enhanced transglycosylation activity. N171A, Y205F and Y299F mutants were expressed as soluble proteins and purified to homogeneity. The hydrolysis activity was detected using ribonuclease B as the substrate [2] and the transglycosylation assay was performed according to the chitinase-coupled assay method [17], [25]. In the chitinase-coupled assay, the transglycosylation product, Man_9_GlcNAc_2_-4-methylumbelliferyl was further hydrolyzed to release a fluorophore, 4-methylumbelliferone (4MU). The fluorescence signal can be correlated to the formation of transglycosylation product. As expected, the mutation N171A completely abolished all enzymatic activity of Endo-A (Table 1). These results are similar to those previously reported for a corresponding residue, N175, in Endo-M, indicating an asparagine at this position is critical for the activity [17]. In the structure of Endo-A in complex with Man_3_GlcNAc-thiazoline, N171 is hydrogen bonded to the thiazoline nitrogen - mimicking the ability of the asparagine to orient the acetamido group for a nucleophilic attack on the anomeric carbon (supplementary Figure S3). Mutant Y205F exhibited reduced hydrolysis activity with an increase in transglycosylation yields (Table 1, Figure 5). Although the transglycosylation activity was similar to that of the wild type Endo-A in 30 minutes, the yield of the transglycosylation product for Y205F is twice as that of the wild type Endo-A after 120 minutes under similar conditions of the assay (Figure 5). A similar mutation in Endo-M (Y217F) was shown to decrease the hydrolysis activity and increase the transglycosylation activity. Analysis of the structure of Endo-A reveals that the Y205F mutation probably results in loss of a potential nucleophile, H_2_O846, which is hydrogen bonded to the hydroxyl oxygen of Y205. Interestingly, another mutation of Endo-A involving a tyrosine, Y299F, showed 3 times higher transglycosylation activity than the wild type Endo-A, while the hydrolysis activity remained unchanged (Table 1). The hydroxyl group of Y299 is forming a hydrogen bond with the oxygen atom of the C2 acetamido group of GlcNAc-Asn (supplementary Figure S4). A loss of the hydroxyl group in the Y299F mutation probably helps release the product faster resulting in an increase in the transglycosylation rate. The transglycosylation product yield for Y299F was 3 times of that of the wild type Endo-A (Figure 5).

**Table 1:** Hydrolysis and transglycosylation activities of Endo-A mutants.

Mutant	Specific hydrolysis activity^a^	Percentage of specific hydrolysis activity	Specific transglycosylation activity^a^	Percentage of specific transglycosylation activity
	mg min^−1^ mg^−1^	%	µmol min^−1^ mg^−1^	%
Wildtype	21.1	100	1.72	100
N171A	ND^b^	-	0.01	0.53
Y205F	9.39	44	1.61	94
Y299F	21.4	101	5.11	297

a The hydrolysis activity was determined using Ribonuclease B as the substrate, the transglycosylation activity was measured using a chitinase-coupled assay, in which Man_9_GlcNAc_2_Asn-Fmoc was used as the donor substrate and 4MU-GlcNAc as the acceptor.

b ND, not detected.

## Discussion

### Mechanism of hydrolysis and transglycosylation

**Figure 6:**
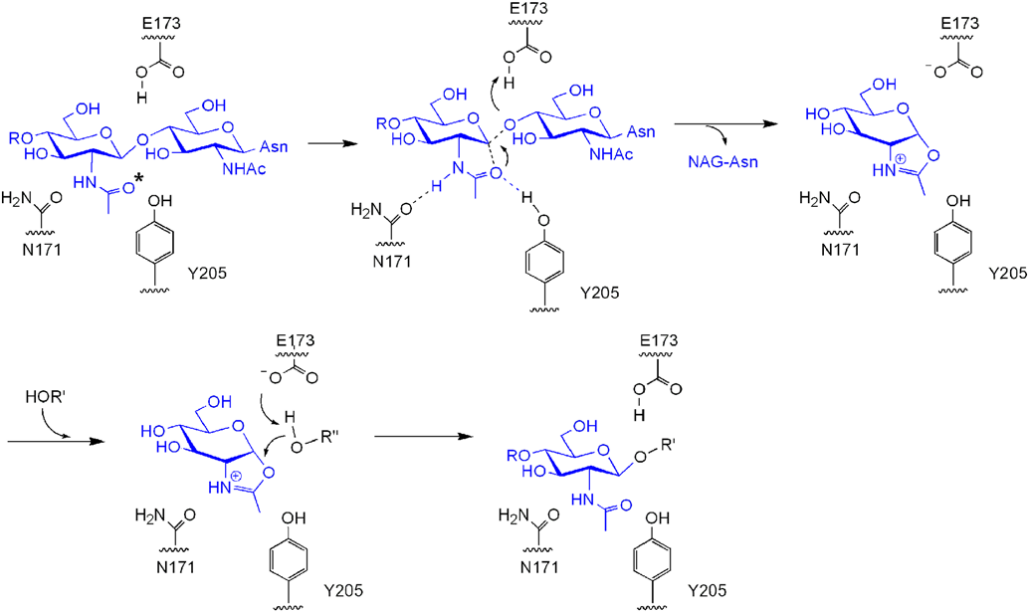
Mechanism of Endo-A mediated catalysis. (A) Substrate surrounded by critical amino acids - N171, E173 and Y205. The nucleophilic oxygen (O) is marked with *. (B) Intramolecular nucleophilic attack. (C) Formation of an oxazoline ion intermediate. (D) Second nucleophilic attack on the intermediate. (E) Synthesis of a new glycosidic bond. R' could be a GlcNAc molecule of an acceptor during transglycosylation.

Hydrolysis in Endo-A is driven by the 2-acetamido component, which acts as a nucleophile in a substrate assisted hydrolysis mechanism. Analysis of the structure of the binary complex of Endo-A with Man_3_GlcNAc-thiazoline reveals that E173 is well positioned to carry out the protonation of the anomeric carbon (Figure 4A, 6). This critical glutamic acid is highly conserved and is observed in all members of the GH85 family of hydrolases. The side chain oxygen of N171 is forming a hydrogen bond at a distance of 2.6 Å with the nitrogen of the C2-acetamido group of the substrate. This is critical for maintaining the correct orientation of the substrate and may help stabilize the oxazoline ion intermediate during substrate assisted catalysis. The oxazoline ion intermediate is subject to further hydrolysis by a water molecule likely to be activated by the hydroxyl oxygen of Y205 or transferred to an acceptor substrate (Figure 6). In the structure of ligand-free Endo-A, H_2_O846 is interacting with Y205 and could fulfill the role of a potential nucleophile. In addition, the hydroxyl oxygen of Y205 is in close proximity of the oxygen atom of the C2-acetoamido group. Y217A mutation in Endo-M (equivalent to Y205 in Endo-A) was shown to severely compromise the enzymatic activity previously [17]. The oxazoline ion intermediate is alternatively synthesized via a substrate assisted catalytic mechanism. N171 orients the acetamido group at C-2 position such that it can mount a nucleophilic attack at the anomeric carbon (Figure 6, supplementary Figure S3). In the structure of Endo-A complexed with GlcNAc-Asn, the carboxyl oxygen of E173 is forming a 2.8 Å hydrogen bond with the O4 hydroxyl oxygen of the pyranoside ring of GlcNAc-Asn (Figure 4A). This interaction activates the acceptor, which can now bind the oxazoline ion intermediate. The overall configuration of the newly synthesized β, 1–4 linkage is the same as that of the one that was hydrolyzed from the donor.

### Engineering ENGases

The Y205F mutation of Endo-A carried out in this study resulted in a decrease up to 56% of the hydrolysis activity (Table 1). Although the transglycosylation activity did not change significantly when compared to the wild type Endo-A, the Y205F mutant of Endo-A could accumulate 2.5 times more transglycosylation product. Mutating Y217 in Endo-M (equivalent to Y205 in Endo-A) to any other amino acid inactivated the enzyme [17]. Therefore, a phenyl ring is critical at this position. Interestingly, another mutant of Endo-A - Y299F - exhibited 3 fold more transglycosylation activity when compared to the wild type Endo-A (Table 1). Analysis of the structure of GlcNAc-Asn bound Endo-A shows Y299 is hydrogen-bonded to the GlcNAc pyranose ring (supplementary Figure S4). A loss of the hydroxyl group, possibly disrupts this hydrogen bond and results in a much faster release of the product when compared to the wild type Endo-A. Thus, mutations that create a more hydrophobic environment facilitate the binding of the sugar moiety and promote the transglycosylation and/or the exclusion of H_2_O resulting in lower hydrolysis activity. Such mutations could be used to engineer ENGases with enhanced transglycosylation activity.

Product hydrolysis is a major issue during the synthesis of glycopeptides and glycoproteins, which makes the overall transglycosylation yield low. Perhaps, the ideal solution to address this problem is to develop glycosynthase-like ENGases. However, since ENGases follow a substrate assisted retaining type of mechanism [16] in which the second acidic amino acid that acts as a nucleophile does not participate in the catalysis, it makes the task of engineering the enzyme more difficult. The structure of Endo-A in complex with Man_3_GlcNAc-thiazoline reveals that N171 may be the essential residue that facilitates the intramolecular attack on GlcNAc during substrate assisted hydrolysis to form the 1,2-oxazoline ion intermediate (supplementary Figure S3). Such an intramolecular attack can be avoided by mutating N171 to a non-nucleophilic residue and would provide an opportunity to develop glycosynthase-like ENGases. In this study, N171A mutation of Endo-A abolished hydrolysis activity. Such an enzyme devoid of hydrolytic activity is well suited for carrying out transglycosylation using sugar oxazolines as donors. In a previous study, Endo-M N175A mutant exhibited transglycosylation activity (though the rate was much lower than the wild-type) without detectable hydrolytic activity when sugar oxazoline was used as a substrate [17].

In summary, we have determined the crystal structure of Endo-A, the first representative structure for members of the GH85 family of hydrolases. Insights into the structural basis and catalytic mechanism for the transglycosylation function have been gained by solving the structure in complex with an inhibitor and an “acceptor”. Y299F mutant exhibited 3 folds transglycosylation activity. The results of this study will assist rational engineering of ENGases.

## Materials and Methods

### Protein expression and purification

The 1863-base pair DNA fragment coding the 70 kDa Endo-A from *Arthrobacter protophormiae* was amplified from pGEX-2T/EndoA plasmid by using forward primer: 5′-TAT*CATATG*TCTACGTACAACGGCCCGCTGT-3′ *and reverse primer: 5′-GAT*GGATCCC*TAAAACGAGCCGCTTTTTATGTCG-3′* containing restriction sites *Nde*I and *BamH*I, respectively. The resulting DNA fragment was ligated into vector pET15b (Novagen) and transformed into *E. coli* BL21(DE3) cells. The cells were cultured in LB medium containing ampicillin (100 µg/ml) at 37°C till OD_600 nm_ reached 0.8. The culture was then induced with 0.2 mM IPTG for 20 h at 16°C. Cells were harvested by centrifugation and lysed by sonication in PBS (137 mM NaCl, 2.7 mM KCl, 50 mM Na_2_HPO_4_, 10 mM KH_2_PO_4_, pH 7.4) containing 0.2 mM PMSF. The lysate was clarified by centrifugation (30,000× g) for 30 min at 4°C. The supernatant was applied to Ni-NTA resin gravity column (Qiagen) that had been previously equilibrated with PBS. The column was first washed with 100 ml PBS followed by washing with 100 ml PBS containing 10 mM imidazole, and finally eluted with PBS containing 500 mM imidazole. After buffer exchange, His-tag was cleaved by thrombin treatment. Uncleaved protein was removed by Ni-affinity chromatography. Endo-A was further purified using Hitrap Q FF ion-exchange chromatography column (Amersham) equilibrated with buffer A (20 mM Tris-HCl, pH 8.0). After rigorous washing with buffer A, bound Endo-A was eluted using a linear gradient of 0 to 1 M NaCl in buffer A. Fractions containing the protein were pooled, concentrated and loaded on Superdex G200 size exclusion chromatography column (Amersham) equilibrated with 20 mM Tris-HCl, pH 8.0, and 200 mM NaCl. Fractions containing the protein were pooled and concentrated to 15 mg/ml before setting up crystallization trials. Se-Met labeled and methylated Endo-A [26] were purified using the same procedure.

### Crystallization and data collection

Crystallization screening was carried out using commercially available sparse matrix screens - Wizard I, II and III (Molecular Dimensions). Trials for native and methylated protein were set up in 2 µl hanging drops containing equal amounts of protein and mother liquor equilibrated over 300 µl of reservoir solution. After 3 days of incubation at 16°C, the native and methylated protein crystallized in a mother-liquor solution containing 80 mM cacodylate, pH 6.5, 14% (w/v) PEG 8000, 160 mM calcium acetate and 20% (v/v) glycerol. After optimization of the initial crystallization condition, crystals for data collection were grown using 100 mM HEPES (pH 7.2) or 100 mM sodium acetate (pH 4.4), 14% (w/v) PEG 8000, 160 mM calcium acetate and 20% (v/v) glycerol. The crystals used for Se-SAD data collection were grown under similar conditions and appeared in one week. Endo-A crystals were soaked with Man_3_GlcNAc-thiazoline or GlcNAc-Asn at 16°C for 2 h before collecting data. A pinch of the substrate powder was directly added to the crystallization drop for soaking experiments. Crystals of the 70 kDa Endo-A belonged to P1 space group with four molecules of the protein in the asymmetric unit. The calculated Matthews coefficient was 2.34 Å^3^/Dalton with a solvent content of 47%.

Crystals were flash frozen in liquid nitrogen prior to data collection. Data sets for the methylated Endo-A native crystals were collected using a Rigaku FRE CuKα rotating anode X-ray source and RAXIS-IV^++^ detector. Although the crystals of methylated Endo-A diffracted well beyond 2.0 Å resolution, due to the limitation of the unit cell parameters and the size of the X-ray beam and detector, the data sets were collected to 2.0 Å resolution only. The data sets for crystals of non-methylated native Endo-A, selenomethionine labeled Endo-A and the two different complexes of Endo-A were collected at Se's peak wavelength (0.9794 Å) using a Quantum-315 CCD area detector at beamline 19-ID and CCDSBC2 detector at 19-BM, Advanced Photon Source, Argonne National Laboratory. All data sets were collected at cryogenic temperature. The data were indexed, integrated and scaled using HKL2000 [27]. The crystals for methylated protein, selenomethionine derivatized and non-methylated native crystals diffracted X-rays to 2.0 Å, 2.9 Å and 2.3 Å resolution respectively. The crystals of Endo-A in complex with Man_3_GlcNAc-thiazoline and in complex with GlcNAc-Asn diffracted X-rays to 2.5 Å and 3.5 Å resolution respectively. Details of data collection statistics are listed in Table 2.

**Table 2:** Data collection and refinement statistics.

Dataset	1	2	3	4	5
Derivatization	Methylated	Se-Met	Non-methylated native	Endo-A with Man_3_GlcNAc-thiazoline	Endo-A with GlcNAc-Asn
X-ray Source	FRE+ SuperBright™	Advanced Photon Source	Advanced Photon Source	Advanced Photon Source	Advanced Photon Source
Detector	R-AXIS IV^++^	Quantum 315	Quantum 315	CCDSBC2	CCDSBC2
Crystal-to-detector distance (mm)	200	350	350	250	320
Wavelength (Å)	1.54	0.9794	0.9794	0.9794	0.9794
Number of images	360	480	360	400	360
Oscillation width (°)	1.0	1.0	1.0	1.0	1.0
Space group	P1	P1	P1	P1	P1
Unit cell parameters *a* (Å) *b* (Å) *c* (Å)	*a* = 79.89*b* = 78.97*c* = 117.27*α* = 84.23*β* = 80.77*γ* = 64.01	*a* = 81.72*b* = 82.85*c* = 114.39*α* = 102.58*β* = 92.30*γ* = 106.26	*a* = 81.28*b* = 82.91*c* = 114.69*α* = 103.01*β* = 91.78*γ* = 106.05	*a* = 78.33*b* = 79.27*c* = 117.04*α* = 80.51*β* = 83.84*γ* = 64.33	*a* = 81.73*b* = 82.80*c* = 115.11*α* = 102.77*β* = 91.72*γ* = 106.71
Resolution range (Å)	20.0–2.0 (2.07–2.0)	50–2.9 (3.0–2.9)	50.0–2.3 (2.38–2.3)	50.0–2.5 (2.54–2.5)	50.0–3.5 (3.65–3.5)
Completeness (%)	67.9 (10.7)	98.4 (98.7)	96.1 (83.4)	95.9 (87.5)	99.1 (99.0)
Total measured reflections	446903 (6760)	430591 (38483)	310849 (26427)	177674 (7457)	130965 (6864)
Unique reflections	116494 (1827)	85254 (8366)	121309 (10571)	86368 (3925)	34434 (1760)
Redundancy	3.8 (3.7)	5.1 (4.6)	2.6 (2.5)	2.1 (1.9)	3.8 (3.9)
Rsym (%)	0.046 (0.175)	0.098 (0.338)	0.036 (0.391)	0.065 (0.254)	0.095 (0.294)
Mean I/σ (I)	46.47 (10.04)	22.05 (4.54)	24 (1.75)	26.90 (2.57)	23.07 (6.33)
Refinement					
Resolution limits (Å)	19.86–2.00			35.4–2.5	
Reflections used	110656			86282	
Number of refined atoms	20628			19782	
Rwork (Rfree, %)	20.30 (25.14)			22.06 (26.37)	
Overall figure of merit	0.824			0.795	
Overall B_Wilson_ (Å^2^)	21.6			36.37	
Protein atoms	18738			18722	
Ligand atoms	1 Mg			4 Man_3_-GlcNAc thiazoline	4 GlcNAc-Asn
	2 Glycerols				
	4 Ca				
	1 PO_4_				
Solvent atoms	1875			872	
Bond RMSD lengths (Å)/angles (°)	0.007/1.027			0.024/1.573	
Mean B value (Å^2^)	22.04			36.84	
All-atom clash score	10.37			16.19	
Ramachandran favored/allowed/outliers (%)	97.05/2.86/0.09			96.96/3.04/0.00	

The numbers in parentheses represent values for the highest resolution shell.

a *R*_sym_ = Σ|*I*_i_−〈*I*〉|/Σ*I* where *I*_i_ is the intensity of the *i*th observation and 〈*I*〉 is the mean intensity of the reflections.

b *R*_work_ = Σ||*F*_obs_|−|*F*_calc_||/Σ|*F*_obs_| where *F*_calc_ and *F*_obs_ are the calculated and observed structure factor amplitude, respectively.

### Structure determination and refinement

ShelxD [28] was used to locate 36 out of 44 selenium sites. The initial phasing was carried out using Oasis [29] and Solve/Resolve [30] using the 2.9 Å Se-SAD data. The experimental phases were transferred and extend to a 2.3 Å non-methylated native data set using DMmulti's 8-fold averaging feature between two crystals [31]. The Autobuild feature in Phenix [32] was able to automatically trace 70% of the residues using the non-methylated native data. The model was manually improved using program Coot [33] to 80% completion before it was used as a MR model to be replaced into the 2.0 Å methylated native data set. The model was further improved after several cycles of Arp/Warp [34] water addition combined with manual building. In order to avoid the NCS correlation artifact during the refinement, the thin-shell technique [35] was used in picking 5% of the reflections as test set for Rfree calculation. The NCS restraints were imposed at all stages of refinement. The refinement was carried out with CNS' simulated annealing [36] and Refmac [37] alternately using the 2.0 Å methylated native dataset and resulted in Rwork value of 20.30% and Rfree value of 25.14% (Table 2). It is worthy to point out that the refinement converged better (lower Rfree and better stereo chemistry) when CNS and Refmac were used alternately than when the refinement was carried out using only one of the refinement programs. It is quite likely that the combined use of the two programs overcomes the systematic error better than the use of individual programs. The quality of the final model was validated using MOLPROBITY [38] with 99.87% of the residues falling either in the most favored or allowed region of the Ramchandran Plot. Structure of Endo-A in complex with its ligands was determined by molecule replacement using the native structure as a search model. Refinement was carried out using Phenix.refine [32].

In the final refined model, four molecules of Endo-A are assembled into two dimers, presumably due to the crystallographic packing effect. A glycerol molecule, possibly originating from the mother liquor used for crystallization, is seen linking the 2 monomers within a dimer by hydrophobic interactions. The site of dimerization is far away from the active centre. In addition, four Ca^2+^, one ion each of Mg^2+^ and PO_4_^−^ are seen in the final model. Electron density for most of the residues from 2–615 was visible for Chains A and B, while most of the amino acids from 3–615 of Chains C and D could be identified in the crystal structure. Backbone density for 14 amino acids – 525:529, 553:555 and 589:594 from China A, 13 amino acids – 525:526, 566:568 and 587:594 from Chain B, 13 amino acids – 525:526, 542:543, 553:554, 569, and 588:593 from Chain C, and 23 amino acids – 527:528, 543:545, 553:555, 567:569 and 584:595 from Chain D could not be traced.

### Synthesis of the Man_3_GlcNAc-thiazoline

The Man_3_GlcNAc-thiazoline inhibitor was synthesized as described previously [22].

### Site-directed mutagenesis

Plasmids containing Endo-A N171A, Y205F and Y299F mutants were constructed using the QuikChange Site-Directed Mutagenesis kit (Stratagene). The Endo-A-pET15b construct (described in protein expression and purification) was used a template for the mutagenesis. The primers used for mutagenesis were designed as follows: N171A_F: 5′- GACGGCTGGTTTATTGCCCAAGAAACAGAAGGG-3′ and N171A_R: 5′- CCCTTCTGTTTCTTGGGCAATAAACCAGCCGTC-3′ for mutant N171A; Y205F_F: 5′- GCACATCATGTGGTTTGACTCGATGATTG-3′ and Y205F_R: 5′- CAATCATCGAGTCAAACCACATGATGTGC-3′ for mutant Y205F; Y299F_F: 5′- CATCACTCGGGTTATTCCGTCCAGATTGGGC-3′ and Y299F_R: 5′- GCCCAATCTGGACGGAATAACCCGAGTGATG-3′ for mutant Y299F. The entire length of the genes were sequenced to verify the mutations and to allow elimination of the constructs with unwanted secondary mutations. Expression and purification of mutants were carried out in the same way as for the wild-type enzyme. Homogeneity of each purified enzyme was confirmed by SDS-PAGE and the concentration of each protein was determined using a protein estimation kit (Bio-rad).

### Activity Assays

The hydrolysis activity of Endo-A and the mutants was assayed using ribonuclease B (Sigma-Aldrich, USA) as the substrate. The reaction mixture was composed of 100 µg of ribonuclease B and suitably diluted enzymes in an acetate buffer (50 mM, pH 6.0, total volume: 100 µl). After incubation for 5 min at 37°C, the reaction was terminated by the addition of 20 µl of 10% trichloroacetic acid. 20 µl of the reaction mixture was analyzed directly by high performance liquid chromatography (HPLC) (Shimadzu, Japan) using a reversed-phase column (Supelco Discovery BIO wide pore C18, 15 cm×4.6 mm). The column was eluted by a gradient of solvent A (water, 0.1% trifluoroacetic acid) and solvent B (acetonitrile, 0.1% trifluoroacetic acid). The gradient ran from 25% B to 30% B over 30 min with a flow rate of 0.5 ml/min. Ribonuclease B and the hydrolysis product, deglycosylated Ribonuclease B, were separated and detected by UV-photometry at 254 nm.

Transglycosylation activity was measured according to the method described previously [25]. Briefly, the 50 µl reaction mixture was composed of 1 mM *N*-glycan Man_9_GlcNAc_2_Asn, 2.5 mM 4MU-GlcNAc, 12% dimethyl sulfoxide, 50 mU of chitinase from *S. griseus*, 0.25 µg enzymes and 50 µl acetate buffer (50 mM, pH 6.0). The reaction was incubated at 37°C. Aliquots (5 µl) were taken at pre-determined time and added to 200 µl glycine buffer (150 mM, pH 10.5) in a Griener 96-well micro titer plate to quench the reaction. Fluorescence was measured on a micro plate reader (Flex Station 3, Molecular Devices, USA) (excitation, 355 nm; emission, 460 nm).

### Accession numbers

Coordinates and structure factors for the native and binary complex of Endo-A with Man3GlcNAc-thiazoline have been deposited in the Protein Data Bank (PDB) under the accession code 3FHA and 3FHQ, respectively. The binary complex of Endo-A with GlcNAc-Asn is in the process of being submitted to the PDB.

## Supporting Information

Text S1 Domain 2 and 3 of Endo-A
(0.02 MB DOC)

Figure S1 Cartoon representation of CBM36, PDB code 1UX7 (panel A), and Domain 2 of Endo-A (panel B). Residues 364–385 form secondary structural elements of Domain 3 (Domain 2 and 3 of Endo-A are interdigitated).
(0.28 MB PPT)

Figure S2 Cartoon representation of the Fibronectin III domain of Integrin α6β4, PDB code 1QG3 (panel A), and Domain 3 of Endo-A (panel B). Electron density for residues 353–355 and 589–594 was missing.
(0.26 MB PPT)

Figure S3 N171 is seen hydrogen bonded to the nitrogen atom of thiazoline. This interaction is critical for the correct orientation of the C2 acetamido group and helps stabilize the transition states. Amino acids and the Man3GlcNAc-thiazoline are shown as sticks.
(1.61 MB PPT)

Figure S4 Mutagenesis of Y299 to a Phe increases transglycosylation activity by 3 fold. Y299 is seen hydrogen bonded to GlcNAc-Asn. A Y299F mutation abolishes this hydrogen bond resulting in faster product release. Amino acids and the GlcNAc-thiazoline are represented as sticks.
(0.58 MB PPT)
