# Supplementary material for: Structural Basis and Catalytic Mechanism for the Dual Functional Endo-β-N-Acetylglucosaminidase A
Source: PLoS One. 2009 Mar 2;4(3):e4658. doi: 10.1371/journal.pone.0004658 (PMC2646837; doi:10.1371/journal.pone.0004658)
Supplement: Text S1 — Domain 2 and 3 of Endo-A (0.02 MB DOC) [file pone.0004658.s001.doc]

**Supplementary text**

**Domain 2 and 3 of Endo-A**

In addition to the catalytic TIM barrel domain, the structure of Endo-A reveals the presence of two more domains at the C-terminus. These domains have never been reported for Endo-A previously and their function is unknown. The secondary structural elements of these two domains are interdigitated and adopt a β-sandwich fold. A search for structural homologues using MSDFold (http:www.ebi.ac.uk/msd-srv/ssm) revealed that domain 2 could possibly be a CBM (Fig S1) while domain 3 shows similarity to Fn3 (Fig S2). Domain 2 of Endo-A consisting of 148 residues was superimposed over the structure of a Family 30 CBD (PDB code 1WMX) made up of 173 residues. The C_α_ atoms of 130 residues matched with an r.m.s. deviation of 2.7 Å. Similarly, C_α_ atoms of 107 residues of domain 2 of Endo-A matched the C_α_ atoms of a 119 amino acids long CBM36 (PDB code 1UX7) with an r.m.s. deviation of 2.7 Å. The carbohydrate binding site of domain 2 of Endo-A could be identified by superimposition of the carbohydrate moiety of CBM36. The sugar superimposes over a structurally similar region of the domain 2 of Endo-A as seen in the CBM36 (data not shown). The secondary structural elements of domain 3 are topologically similar to the Fn3 domain. Domain 3 of Endo-A is 101 amino acids long. Integrin α6β4 has a tandem pair of Fn3 domains at its cytoplasmic tail. The C_α_ atoms of 72 residues of domain 3 of Endo-A matched with the C_α_ atoms of one of the Fn3 domains of integrin (PDB code 1qg3, 98 amino acids long) with an r.m.s. deviation of 2.3 Å.

CBM and Fn3 domains have been reported for a number of carbohydrate degrading enzymes. Domain 2 of Endo-A is probably a CBM and plays a role in increasing the efficiency and specificity of carbohydrate binding. Domain 3 of Endo-A may be involved in protein-carbohydrate or protein-protein interactions during cell adhesion or events requiring modulation of the cell wall. Further studies are underway to determine the exact role of these domains in Endo-A.
