# Supplementary figures and images for: Structural Basis and Catalytic Mechanism for the Dual Functional Endo-β-N-Acetylglucosaminidase A
Source: PLoS One. 2009 Mar 2;4(3):e4658. doi: 10.1371/journal.pone.0004658 (PMC2646837; doi:10.1371/journal.pone.0004658)

## Slide 1
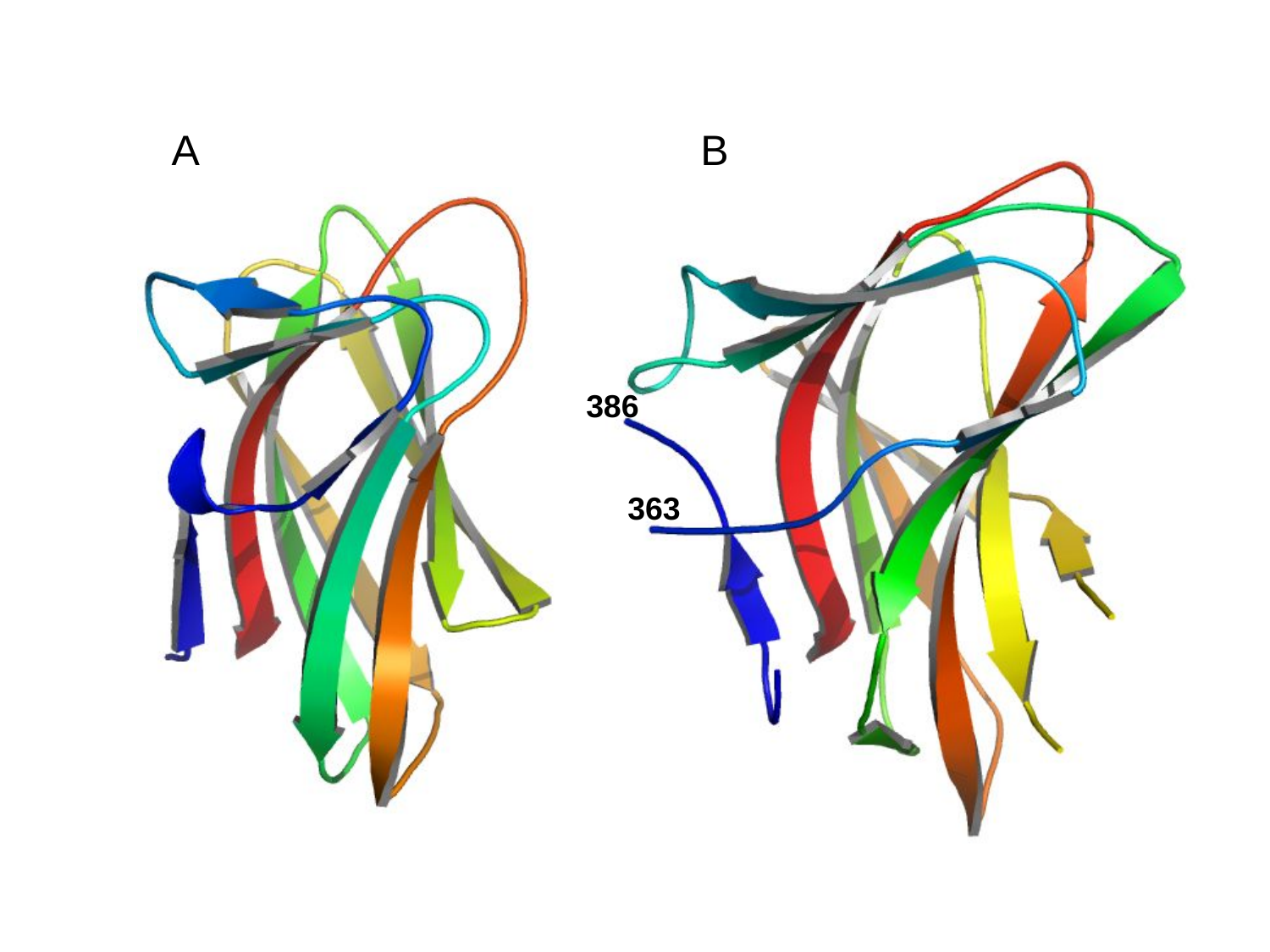

A
B
386
363

Supplement: Figure S1 — Cartoon representation of CBM36, PDB code 1UX7 (panel A), and Domain 2 of Endo-A (panel B). Residues 364–385 form secondary structural elements of Domain 3 (Domain 2 and 3 of Endo-A are interdigitated). (0.28 MB PPT) [file pone.0004658.s002.ppt]

## Slide 1
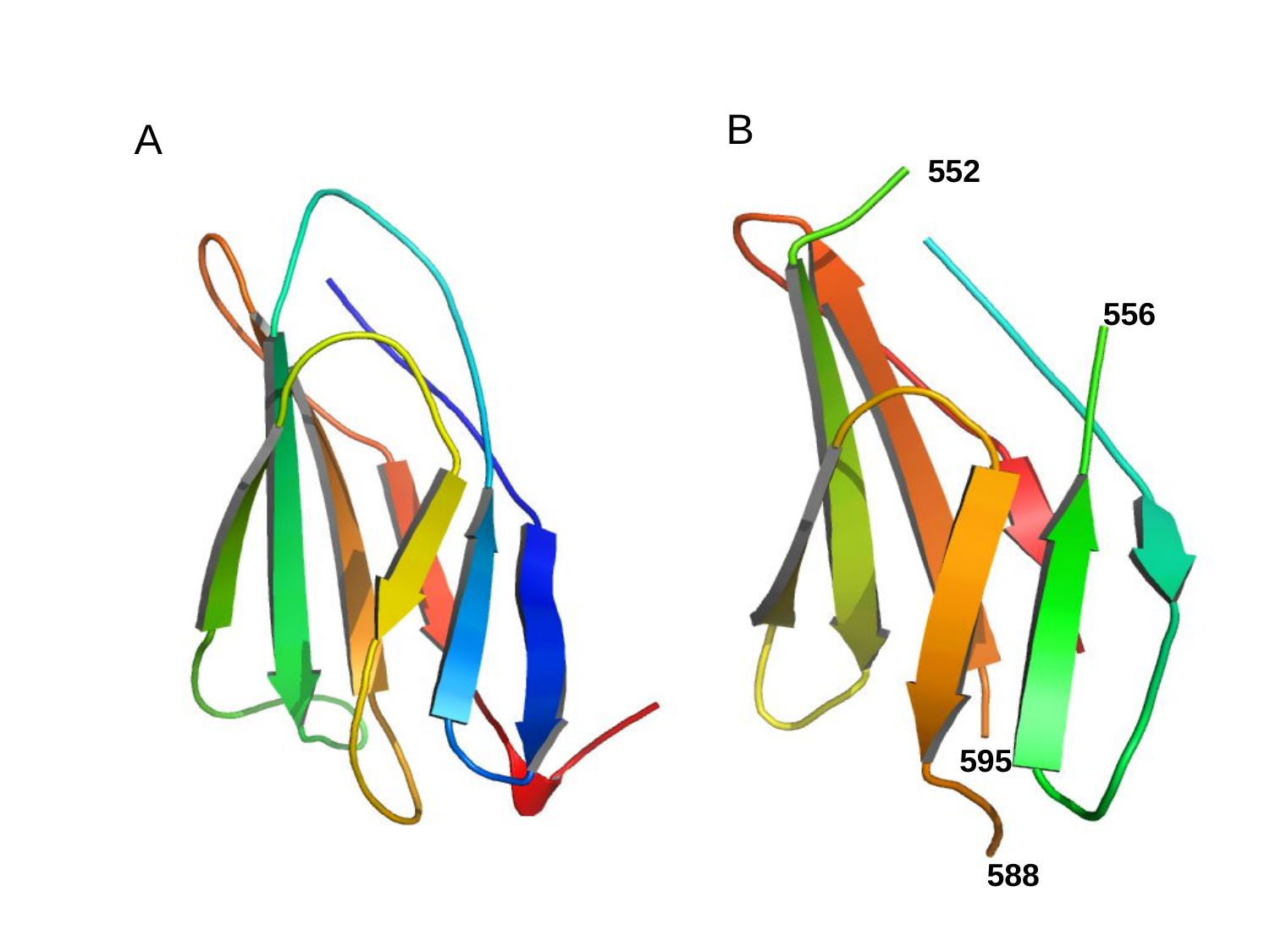

B
A
552
556
595
588

Supplement: Figure S2 — Cartoon representation of the Fibronectin III domain of Integrin α6β4, PDB code 1QG3 (panel A), and Domain 3 of Endo-A (panel B). Electron density for residues 353–355 and 589–594 was missing. (0.26 MB PPT) [file pone.0004658.s003.ppt]

## Slide 1
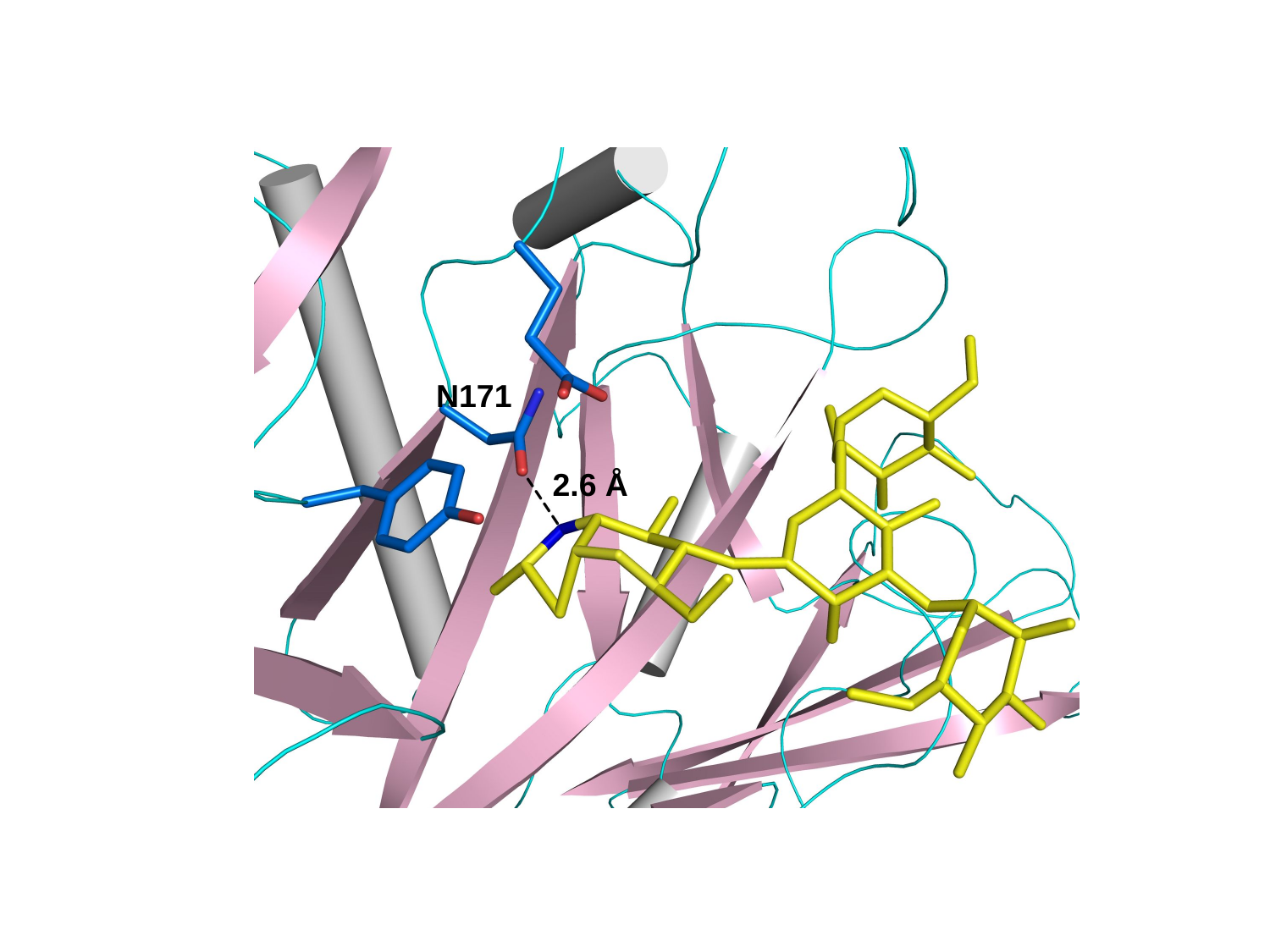

2.6 Å
N171

Supplement: Figure S3 — N171 is seen hydrogen bonded to the nitrogen atom of thiazoline. This interaction is critical for the correct orientation of the C2 acetamido group and helps stabilize the transition states. Amino acids and the Man3GlcNAc-thiazoline are shown as sticks. (1.61 MB PPT) [file pone.0004658.s004.ppt]

## Slide 1
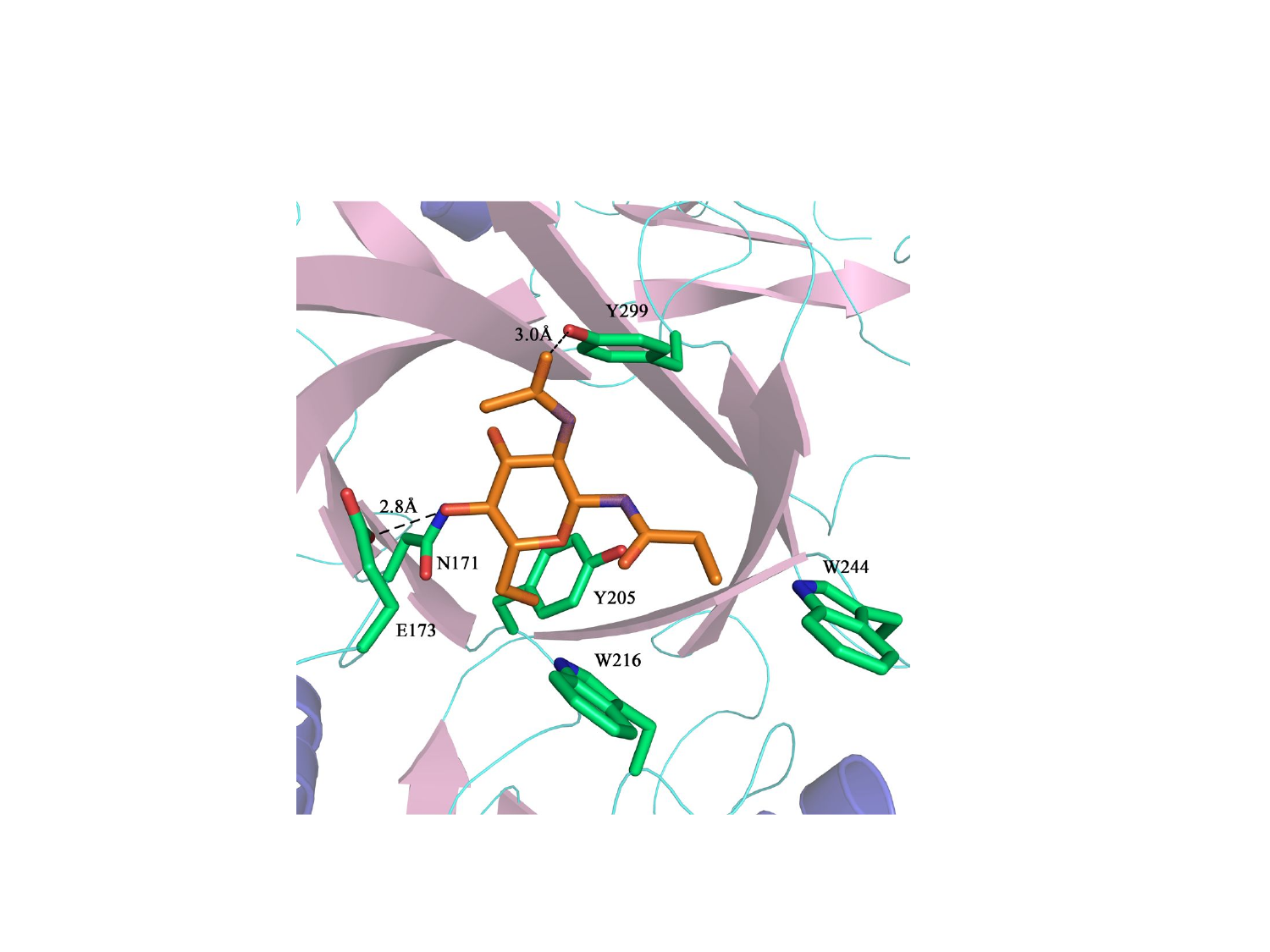

Supplement: Figure S4 — Mutagenesis of Y299 to a Phe increases transglycosylation activity by 3 fold. Y299 is seen hydrogen bonded to GlcNAc-Asn. A Y299F mutation abolishes this hydrogen bond resulting in faster product release. Amino acids and the GlcNAc-thiazoline are represented as sticks. (0.58 MB PPT) [file pone.0004658.s005.ppt]
